# Pre- and Post-Transcriptional Control of HBV Gene Expression: The Road Traveled towards the New Paradigm of HBx, Its Isoforms, and Their Diverse Functions

**DOI:** 10.3390/biomedicines11061674

**Published:** 2023-06-09

**Authors:** Rodrigo A. Villanueva, Alejandra Loyola

**Affiliations:** 1 Centro Ciencia & Vida, Fundación Ciencia & Vida, Santiago 8580702, Chile; aloyola@cienciavida.org; 2 Facultad de Medicina y Ciencia, Universidad San Sebastián, Santiago 7510602, Chile

**Keywords:** hepatitis B virus, viral replication, hepatitis B virus X protein, HBx, isoform protein, viral regulatory protein, mRNA, pre- and post-transcriptional control

## Abstract

Hepatitis B virus (HBV) is an enveloped DNA human virus belonging to the *Hepadnaviridae* family. Perhaps its main distinguishable characteristic is the replication of its genome through a reverse transcription process. The HBV circular genome encodes only four overlapping reading frames, encoding for the main canonical proteins named core, P, surface, and X (or HBx protein). However, pre- and post-transcriptional gene regulation diversifies the full HBV proteome into diverse isoform proteins. In line with this, hepatitis B virus X protein (HBx) is a viral multifunctional and regulatory protein of 16.5 kDa, whose canonical reading frame presents two phylogenetically conserved internal in-frame translational initiation codons, and which results as well in the expression of two divergent N-terminal smaller isoforms of 8.6 and 5.8 kDa, during translation. The canonical HBx, as well as the smaller isoform proteins, displays different roles during viral replication and subcellular localizations. In this article, we reviewed the different mechanisms of pre- and post-transcriptional regulation of protein expression that take place during viral replication. We also investigated all the past and recent evidence about HBV HBx gene regulation and its divergent N-terminal isoform proteins. Evidence has been collected for over 30 years. The accumulated evidence simply strengthens the concept of a new paradigm of the canonical HBx, and its smaller divergent N-terminal isoform proteins, not only during viral replication, but also throughout cell pathogenesis.

## 1. Introduction

### 1.1. Hepatitis B Virus and Its Replicative Cycle

**Figure 1:**
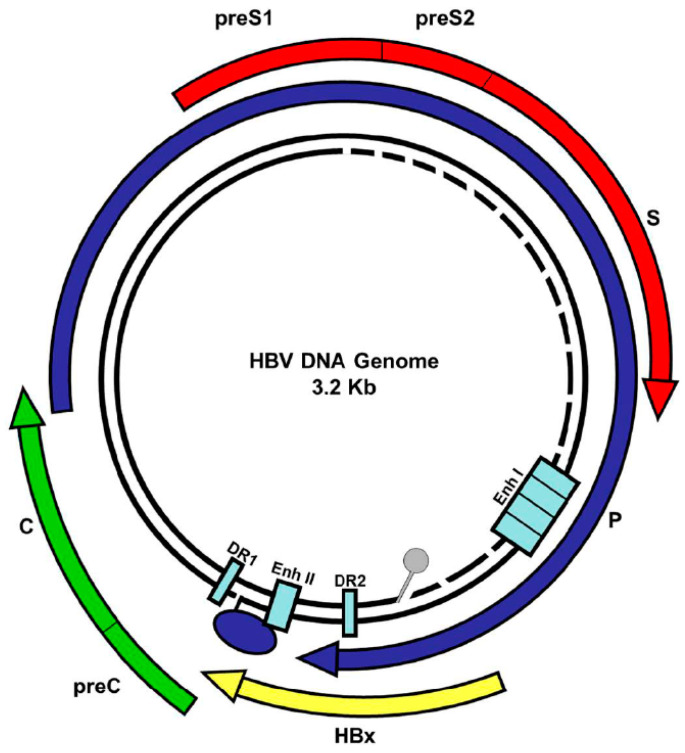
Hepatitis B circular genome. The HBV genome is a partially double-stranded, relaxed, and circular DNA of about 3.2 kb. The genome contains four overlapping reading frames encoding for the viral envelope or surface (pre-S1/pre-S2/S, red arrow), core (pre-core/core) (green arrow), polymerase (blue arrow), and HBx protein (yellow arrow). As shown, the DNA genome contains two direct repeats (DR1 and DR2), two enhancers (EnhI, EnhII), and four promoter regions that regulate protein expression. Viral P protein is covalently bound to the genome (blue) and an RNA primer is bound to the negative strand (gray).

The human hepatitis B virus (HBV) is a membrane-enveloped DNA virus classified as a member of the viral family *Hepadnaviridae*. Its main distinguishable characteristic is the replication of its genome through a reverse transcription process. As shown in Figure 1, the HBV circular genome encodes only four overlapping reading frames, encoding for the main canonical proteins named core, P, surface, and X (or HBx protein) [1].

However, pre- and post-transcriptional gene regulation diversifies the full HBV proteome into diverse isoform proteins. The viral genome is an incomplete double-stranded, relaxed, circular DNA (rcDNA), composed of two complementary strands of distinct extents. The approximately 3.2 kb long strand is the coding (-) strand, while the length of the complementary (+) strand varies from 50% to 100% of the (-) strand, Figure 1 [1]. HBV is distributed into four mains serological serotypes (adr, adw, ayr, ayw) and ten different genotypes (varying in no less than 8% of their nucleotide sequences), referred to as genotypes A to J. Different HBV genotypes display distinct geographical distribution, prevalence, disease evolution, and treatment response [2]. For instance, Latin America shows a high prevalence of the native HBV genotypes F and H which are both autochthonous. HBV genotype F (e.g., HBV subgenotype F1b, GenBank KM233681.1, 3215 bp [3,4]) is related to early, frequent, and quick progression to hepatocellular carcinoma (HCC) [5,6,7,8], while genotype H infection is associated with low endemicity, low viral load, few cases of acute and chronic liver diseases, and a low prevalence of HCC [9,10,11]. Additionally, occult hepatitis B infection is highly prevalent in HBV genotype H infection [11,12,13]. Unfortunately, the molecular determinants that differentiate the infection courses of these two genotypes are unknown.

In general, as mentioned, viral infection displays parenteral transmission and can cause both acute (self-limited) and chronic infections. More than 50% of long-term, persistent, chronic HBV infections are caused through mother-to-child transmission, which occurs either vertically or horizontally, by exposure to infectious blood or body fluids; however, HBV can also be spread sexually [14].

A small number of mammals and birds are infected by viruses in this family. These viruses all include circular DNA genomes such as the woolly monkey hepatitis B virus (WMHBV; GenBank AF046996.1, 3179 bp), the woodchuck hepatitis B virus (WHBV; GenBank AY334075.1, 3308 bp), the ground squirrel hepatitis B virus (GSHBV; GenBank K02715.1, 3311 bp), and the duck hepatitis B virus (DHBV; GenBank K01834.1, 3021 bp) [15]. They all have a limited host range for hepatocytes.

In humans, the attachment of virion particles to the cell surface receptor (sodium taurocholate co-transporting polypeptide, NTCP), which is a transmembrane protein expressed in hepatocytes, initiates the viral life cycle, via endocytosis. Nucleocapsids harboring viral rcDNA are released inside hepatocyte cytoplasm after particle penetration at the plasma membrane, then soon reach the cell nucleus [16]. Through several enzymatic reactions, rcDNA is repaired and transformed into a viral intermediate molecule known as covalently closed circular DNA (cccDNA), thus producing an episomal mini chromosome structure [17]. All viral transcripts, including the 3.4 kb pre-genomic mRNA (pg-mRNA) that is co-linear with the genome, are generated by transcription using the nuclear cccDNA as a template [1]. The nuclear synthesis of the pg-mRNA transcript, which is then exported to the cytoplasm, is crucial for two distinct processes: (i) it serves as a template for the translation of both the viral reverse transcriptase (P protein) and core protein and (ii) the reverse transcription process is started using the pg-mRNA as a template when these two viral proteins are produced in the cytoplasm [18]. Both the epsilon stem–loop region of the pg-mRNA and the HBV P protein are covalently bound and combine with the capsid protein to form an immature viral capsid structure [19]. Thus, the epsilon stem–loop plays a key role during particle formation. The reverse transcription of pg-mRNA into rcDNA occurs inside the capsid by HBV P protein and produces a cytoplasmic mature viral capsid. A mature capsid can either re-enter the nucleus to maintain the nuclear pool of cccDNA or be enveloped by HBV surface proteins on endoplasmic reticulum (ER) membranes for virion egress, which depends on functions of the so-called multivesicular body (MVB) pathway [20]. Moreover, double-stranded linear DNA (DSL-DNA), a minor and different viral DNA species produced by reverse transcription, can also be released as virion DNA and guided to enter the nucleus to generate cccDNA [21]; however, DSL-DNA is commonly randomly integrated into the host genome through the non-homologous end-joining pathway, utilizing double-stranded DNA breaks in the host DNA target [22,23]. These integration events may accelerate the development of HCC by causing chromosomal instability and insertional mutagenesis of HBV genes, amongst other genetic alterations [24]. The integrated HBV genome, in contrast to cccDNA, does not generate functional pg-mRNA for viral replication but may act as a second source for surface proteins, mRNA transcription, and HBsAg production [25].

### 1.2. HBV Genes and Translation of Viral RNA Transcripts into Viral Isoform Proteins

Due to the utilization of several internal AUG initiation codons in the same open reading frame, alternative translation initiation is a mechanism by which a single mRNA molecule causes the translation of isoform proteins with different N-termini [26,27]. As a result, this mechanism modifies protein function and/or subcellular localization, which expands the proteome [26]. Although certain ribosomes may bypass the start codon and carry on scanning, the translation initiation mechanism in eukaryotes generally follows the scanning ribosome model, in which the ribosome scans the mRNA from the 5′-end, and initiates translation at the first AUG codon detected [28,29,30]. This process is known as “leaky scanning” and is determined by the sequence context of each AUG. The general regulation of the translation initiation comes from Kozak’s consensus sequence: (A/G)XXAUGG (start codon underlined), in which the −3(A/G) and +4G are the first and second most important bases for efficient initiation, respectively (+1 is the first base of the start triplet) [28,29,30]. In the literature, the number of protein isoforms whose expression is regulated by alternative translation initiation is progressively increasing [26,31,32].

Importantly, we utilized the web server https://atgpr.dbcls.jp/ (accessed on 20 March 2023) [33], which is software for identifying initiation codons in cDNA sequences according to Kozak’s consensus rule to analyze the potential whole HBV proteome arising by alternative translation initiation from each viral reading frame. Full prediction results are presented in Supplementary Table S1, where the canonical HBV reading frames are shown in bold and reliability is the probability that the prediction will be achieved correctly. Records will be commented on for each viral reading frame as follows.

The core, polymerase (P), envelope (S), and X canonical viral genes are located within the four broadly overlapping reading frames that make up the circular HBV genome DNA, Figure 1. The basal core promoter (BCP), S1, S2, and X are the four primary promoters that control the expression of the HBV genome, directing the host nuclear transcriptional machinery (RNA polymerase II and transcription cell factors) to initiate transcription at various locations to produce a variety of mRNA species with lengths of 3.5, 3.4, 2.4, 2.1, and 0.7 kb [1,34]. To translate proteins and replicate DNA, viral mRNAs are exported to the cytoplasm. These viral mRNAs are 5′-capped, and all are co-terminated at the same position in a single polyadenylation signal at their 3′-ends, which is a hexanucleotide (HBV DNA nt 1916 to 1921, sequence TATAAA) sequence of the viral DNA [35]. In addition, two enhancer elements (EnhI and EnhII), and the transactivity function of the HBV HBx protein (X gene product) can further stimulate the viral transcription [36].

**Figure 2:**
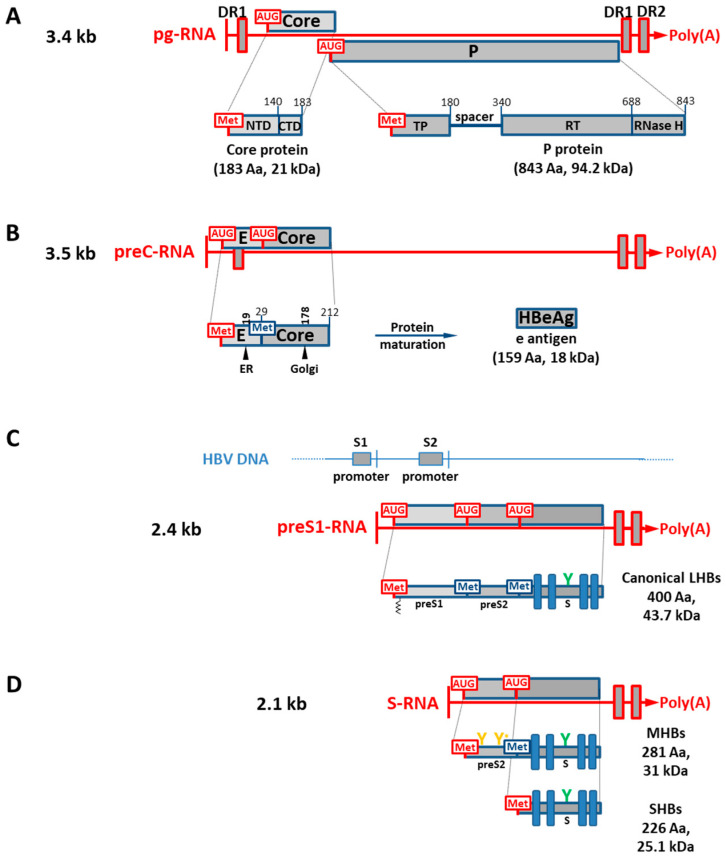
HBV mRNAs and their coding capacity. (**A**) pg-RNA, 3.4 kb. pg-RNA is organized in a bi-cistronic arrangement where the first AUG is encoding for core, and downstream, in a different reading frame, the AUG codes for the P protein. Core and P proteins are shown with their main domain organizations. (**B**) preC-RNA, 3.5 kb. preC protein starts translation upstream to the core’s AUG, but in the same reading frame. Sites for cleavage events (ER and Golgi apparatus) on the immature protein are shown. As a result, HBeAg is a secreted protein. (**C**) preS1-RNA, 2.4 kb. At the top, HBV DNA is shown with the relative locations of S1 and S2 promoters. preS1-RNA is predicted to encode the canonical LHBs, although other AUGs are present in the same reading frame (see the text). LHBs is shown with its domains preS1, preS2, and S. Both preS1 and S domains bear PTMs as shown. (**D**) S-RNA, 2.1 kb. S-RNA is predicted to encode both preS2 and S domains where MHBs and SHBs are produced.

HBV transcriptions of both the largest pre-core RNA (pC-mRNA) and pre-genome RNA (pg-mRNA) transcripts are controlled by the BCP (nt 1740 to 1850) region of the viral genome. The HBV EnhII region is located immediately upstream of BCP (EnhII, nt 1620 to 1740) [37]. HBV BCP/EnhII activity is regulated by different factors, such as CCAAT/enhancer-binding protein (C/EBP), hepatocyte nuclear factor 4 (HNF4), HNF3, and the cell factor named specificity protein 1 (Sp1) [38]. As indicated above, both particle morphogenesis and genome replication depend on pg-mRNA, which also serves as a template for the translation of the viral core and P proteins. These reading frames are structured in a bi-cistronic arrangement, where the P protein is independently translated from pg-mRNA through ribosome “leaky scanning” past the AUG of the core reading frame. Thus, as shown in Figure 2A, the 5′-end of the P cistron’s AUG overlaps with the 3′-end of the core gene [39,40,41].

On the other hand, as shown in Supplementary Table S1, the canonical core reading frame has two internal AUG initiation codons that could function as alternative initiation codons for translation, producing two smaller core isoforms of 13.6 and 10.6 kDa, although core isoform 1 has a higher reliability than that of core isoform 2. Both core isoforms would maintain an intact C-terminal domain, which is the RNA-binding domain (an arginine-rich region) of this HBV capsid protein (Figure 2A) [42]. On the other hand, the reading frame of the P protein bears ten different internal AUG initiation codons, but only two of them might function as initiation codons based on the threshold and their reliability. Thus, P protein would have two smaller isoforms of 41 and 20.8 kDa with somewhat similar reliabilities. In the case of the N-terminal truncated P protein of 41 kDa, this isoform protein would initiate translation within the reverse transcriptase (RT) domain, having an intact YMDD active site motif and RNase H domain, whereas the 20.8 kDa isoform would only contain a complete RNase H domain, Figure 2A [43]. Interestingly, the HIV-1 RT forms functionally active heterodimers where one subunit is the complete RT polypeptide, while the other subunit is a truncated RT polypeptide lacking the RNase H domain, which is lost by a proteolytic cleavage performed by a viral protease [44].

An additional 5′ extension with an AUG at the 5′-end of the pre-core mRNA (pc-mRNA, 3.5 kb) differentiates it from the pg-mRNA [45,46] (Figure 2B). However, the pc-mRNA transcript is believed to only be involved in the expression of the pre-core protein, which is the hepatitis B e antigen precursor (HBeAg or early antigen). As during translation, the pre-core protein translocates to the ER and then to the Golgi apparatus as the protein is processed for maturation. A 36 kDa dimeric HBeAg protein is then released after both amino- and carboxyl-terminal cleavage events (Figure 2B) [47,48]. HBeAg is a secreted protein which is essential for establishing persistent/chronic infection through the formation of immunological tolerance, but it is not necessary for viral replication [49,50]. Consistent with the translational information contained in the pc-mRNA, the canonical isoform, as well as two smaller core isoforms, would also be translated from this transcript, although with significantly less reliability than that of predominant HBeAg protein, as shown in Supplementary Table S1.

A 2.4 kb mRNA species transcribed under the control of the hepato-specific promoter SI (or SPI), encoding the canonical and complete preS-S reading frame (the HBV canonical surface gene), is thought to be mostly translated into L protein (Figure 2C) [51,52]. The promoter SI/SPI (nt 2750 to 2840) is situated upstream of AUG1, and it seems to be regulated by transcription factors such as Oct-1, HNF1, and HNF3 [38]. This reading frame bears two additional initiation codons, AUG2 and AUG3, which would both favor the expression of the canonical large surface proteins (LHBs) as well as two smaller isoform proteins named middle surface proteins (MHBs) and small surface proteins (SHBs), at one of three in-frame initiation codons from the entire reading frame (Figure 2C), as shown in Supplementary Table S1. These three surface proteins all share a common C-terminal end which corresponds to the S isoform protein (SHBs). Thus, by alternative translation initiation at one of the specific AUGs in the HBV surface gene, three divergent N-terminal isoform envelope proteins can be generated [53]. Due to their unique N-termini, these surface proteins exhibit various functional differences during particle assembly and HBV infection (Figure 2C,D) [41].

There is an intragenic promoter within the HBV canonical surface gene called SII/SPII (nt 2960 to 3150). It is located within the preS-S reading frame, and it regulates transcription of a 2.1 kb mRNAs with different 5′-ends either upstream or downstream of AUG2 (Figure 2C,D) [54,55]. This SII/SPII promoter seems to be upregulated by the cellular nuclear transcription factor Y (NF-Y), CCAAT-binding factor (CBF), Sp1, and NF1 [38]. It is believed that the 2.1 kb mRNA segment, which includes AUG2, is translated into both MHBs and SHBs [52,56] as indicated by Supplementary Table S1. The major (SHBs) protein is produced by translation initiation at AUG3 (p24 and gp27); initiation at AUG2 adds 55 amino acids to the N-terminus (named preS2 region, Figure 2C,D), producing the middle (MHBs) protein (p30, and gp33 and gp36). Interestingly, the translation of a smaller isoform protein of 17.2 kDa and 152 residues at the C-terminal domain would be favored from the S-mRNA transcript (Supplementary Table S1). Thus, the translation of the HBV envelope gene is regulated by both the alternative translation and transcription initiations, which both generate critical functional diversity of the HBV surface proteins.

### 1.3. HBV Viral Isoform Proteins Generated by RNA Splicing

mRNA splicing is a post-transcriptional process that allows the translation of a cytoplasmic, mature mRNA into protein [57]. This process takes place by removing the intervening, non-coding nucleotide sequences of genes (introns) from the nuclear, immature pre-mRNA molecules, then ligating the protein-coding nucleotide sequences (exons) [58]. In addition, a process called alternative splicing enables several exon combinations to be included in the final mature mRNA molecule, resulting in various distinct isoforms of the same protein that are all encoded by the same DNA gene [59,60].

Following infection, various mammalian viruses exploit the cell splicing machinery to carry out their replication cycles, and so refine protein diversity [61,62]. Members of the *Retroviridae*, *Adenoviridae*, *Herpesviridae*, and *Papillomaviridae* families and lentiviruses such as HIV [61,63,64,65] are only a few viral families that use host splicing machinery. Numerous studies have discussed the splicing processes of the HBV pg-mRNA transcript, which also serves as a template for genome replication as indicated earlier [66]. Splicing activities on subgenomic viral mRNAs have also been described. Other members of the *Hepadnaviridae* family, such as WHBV and DHBV, encode several viral RNAs going through alternative splicing [67,68]. Importantly, alternative splicing of HBV pre-genomic RNAs generates at least twenty unique splice variants [69]. The preservation of the epsilon stem–loop region at the 5′-end of spliced RNA isoforms, however, indicates a potential for particle packaging and usage as a reverse transcription template. Thus, nearly every one of these spliced forms would produce defective genomes and viral particles, which can be found in the blood of HBV-infected patient samples [70,71]. Spliced RNAs do not appear to be essential for viral replication, but mounting evidence points to a role in liver pathogenesis [70].

From HBV-spliced RNAs, several potential shortened or original isoform proteins could be produced.

### 1.4. Hepatitis B-Spliced Protein (HBSP; 12.3 kDa)

**Figure 3:**
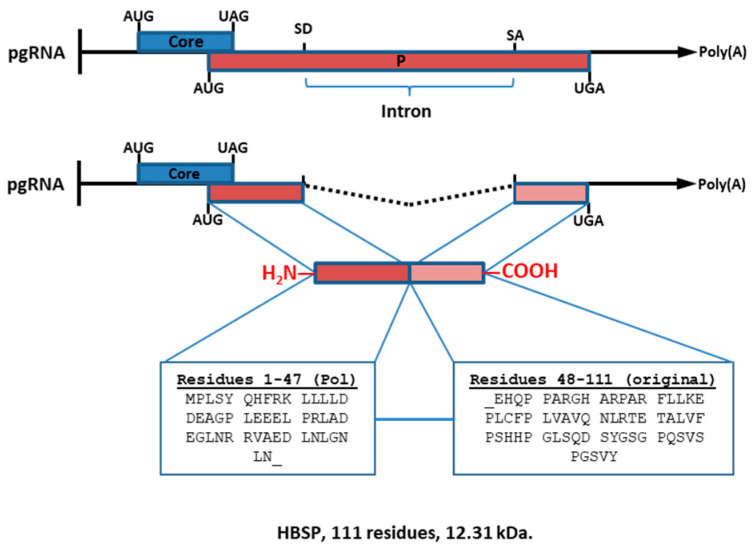
Hepatitis B-spliced protein, HBSP. HBSP is produced from a single splicing event, and it is encoded by the 2.2 kb sp1 RNA molecule of the HBV pg-mRNA transcript. Both core and P reading frames are shown. SD, splice donor site, SA, splice acceptor site, and intron, are shown, located within the P reading frame. During translation initiation, the 47 first residues of P protein are translated, and then the junction changes the reading frame, and so the sequence of the C-terminal half of this protein is unique.

This protein is produced from a single splicing event, and it is encoded by the 2.2 kb singly spliced 1 (sp1) RNA molecule of the HBV pg-mRNA transcript. HBSP is usually identified as the “eighth HBV protein”. The N-terminal amino acids of the HBSP sequence are identical to those of the viral polymerase (47 aa), whereas the C-terminal amino acids (64 aa) are unique because of the splicing event (Figure 3).

The biological role of the HBSP fusion protein is unclear. HBSP has been detected in liver biopsies from chronic patients, and in the sera, where α-HBSP antibodies have also been found [72,73]. The expression of HBSP reduces the apoptosis mediated by Fas by improving the PI3K/Akt signaling [74].

### 1.5. HBV Polymerase-Surface Fusion Protein, HBV P-S Fusion ORF

This glycoprotein is produced from a single splicing event, and it is encoded by the 2.2 kb sp14 RNA molecule corresponding to the viral pg-RNA bearing a deletion of 454 nt. This 43 kDa P-S fusion protein has been identified in extracellular viral particles, cell lysates, and subviral particles. It is believed that, during virion maturation, this fusion protein can replace the role of HBV LHBs [75,76]. Within cells, the HBV P-S fusion protein is localized within the perinuclear area, and it can block HBV genome replication by the suppression of HBsAg secretion. This function might improve immune evasion, implying a role in HBV pathogenesis.

### 1.6. Hepatitis B Double Splicing Protein, HBDSP

This viral protein is produced by a sp7 RNA molecule after a double splicing event takes place. It was first identified in liver HCC biopsy samples, lacking two intron RNA sequences [77,78]. The dimeric activator protein-1 (AP-1) and CCAAT/enhancer-binding protein (C/EBP) family of transcription factors exhibit several transactivating abilities and pleiotropic effects. However, precise mechanisms associated with the regulatory functions of HBDSP and its biological implication in HBV pathogenesis need further investigation.

### 1.7. The Canonical HBx Is a Unique Multifunctional Regulatory HBV Protein

**Figure 4:**
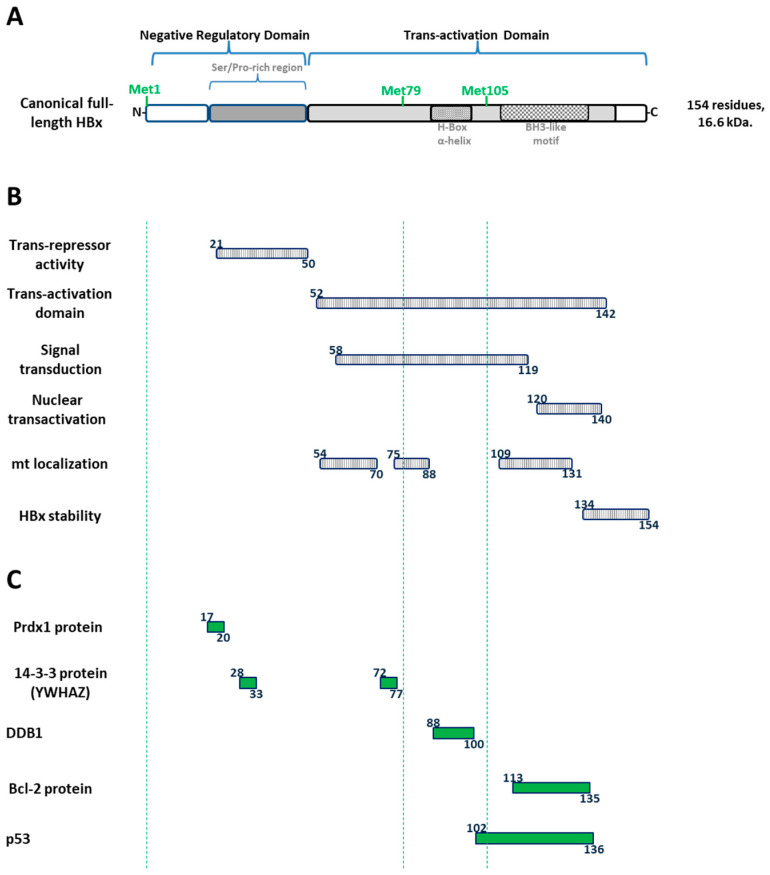
Domain organization of the canonical HBx protein and interacting partners. (**A**) HBx protein is organized into two distinct functional domains. The N-terminal negative regulatory domain (residues 1–50) is composed of a highly conserved N-terminal region and a Ser/Pro-rich region as indicated. The C-terminal region contains the crucial transactivation domain covering the AUG2 (Met79) and AUG3 (Met105). Both the H-box alpha-helix and the BH3-like motif are shown. (**B**) Mapped location of HBx functional regions. Regions of transrepressor activity, transactivation domain, signal transduction, nuclear transactivation, putative mitochondrial localization, and protein stability are shown. (**C**) Mapped interacting partners. Regions of interactions with Prdx1, 14-3-3, DDB1, Bcl-2, and p53 proteins are shown.

Canonical HBx protein corresponds to the smallest gene (465 nucleotides, 154 amino acids, and 16.6 kDa) and it is the only regulatory protein of HBV. The protein is common to all the mammalian members of the *Hepadnaviridae* family, including woodchucks, but absent in avian viruses such as DHBV (e.g., GenBank K01834.1). The HBx protein is essential for viral replication and HBV infection [79,80,81]. The multifunctional HBx protein is organized into two distinct functional domains (Figure 4A) [82,83,84].

The N-terminal domain (residues 1–50) is composed of a highly conserved N-terminal region displaying transrepressor activity (residues 21–50, Figure 4B) [85] as well as a Ser/Pro-rich region apparently required for dimerization (residues 21–50) [83], which is also important for cellular transformation [86]. The C-terminal region is fundamental for HBx functionality as it contains the essential transactivation domain of the protein (residues 52–142, Figure 4B) [82,87,88]. Moreover, within the C-terminal region, further functional studies have shown that residues 58–119 are involved in signal transduction activities [89], while residues 120–140 are involved in nuclear transactivation mechanisms [87,90], and the last 20 residues are involved in HBx stability [91] (Figure 4B). Furthermore, HBx also contains a putative mitochondrial localization domain, and expression of HBx at the mitochondria alters its metabolism [92,93]. The 3D structure of HBx is unknown. While the N-terminus bears a highly conserved but unstructured and disordered region [94], the C-terminal of the protein is more structured [95]. On the other hand, a plethora of HBx-interacting proteins have been described (Figure 4C) [96], targeting its different domains, and so affecting different intracellular pathways. Only several instances of mapped HBx interactions are completely known, and a few of them are described in Figure 4C. HBV HBx protein has been described to interact with peroxiredoxin-1 (Prdx1) protein through its N-terminal/negative regulatory domain, residues 17 to 20 [97]. It has been shown that the adapter 14-3-3 zeta protein interacts with residues 28 to 33 of the N-terminal domain of HBx [98], and also with residues 72 to 77 of the transactivation domain, although this latter complementary interaction has not been empirically proved [98]. The multifunctional DNA damage-binding protein 1 (DDB1) interacts with the transactivation domain, in a segment of residues 88 to 100 of HBx, which forms a promiscuous α-helix, named the H-box [99]. Interestingly, the segment of residues of the H-box of HBx is located between AUG2 and AUG3 (Figure 4C). Moreover, the transactivation domain of HBx through its BH3-like motif (residues 113 to 135), which folds into a short α-helix, is utilized to interact with the anti-apoptotic factor Bcl-2 and activate HBV replication [100]. Finally, tumor suppressor TP53 (p53) is well known to extensively interact with the transactivation domain of HBx, residues 102 to 136 (Figure 4C) [101].

There is no agreement on a unique subcellular localization of the HBx protein since several in vitro analyses and liver biopsy examinations have indicated the protein to be predominantly cytoplasmic [102,103,104]; other studies have determined it to be a nuclear protein [105,106]. This inconsistency is probably because the subcellular localization of the protein is highly regulated by its degree of abundance. Thus, when transfected at high levels, HBx is found in the cytoplasm, whereas when present at low abundance, it is mostly confined to the nucleus [107,108]. Importantly, the HBx protein’s multiple functions may be explained by its distinct nuclear/cytoplasmic and mitochondrial localization [109,110]. HBx can modify the metabolism of mitochondria and alter apoptosis pathways and a variety of signal transduction cascades in the cytoplasm [111,112,113,114,115,116]. In the nucleus, HBx can transactivate different viral and cell promoters [117]. As it is necessary for viral replication [118], a nuclear pool of HBx is required. Although HBx does not directly bind to dsDNA [119], it clearly binds in vitro to ssDNA [120], and HBx’s ability to activate transcription of cellular genes is thought to occur through interactions with nuclear proteins such as transcription factors [83,120,121,122]. Additionally, DNA repair pathways are impacted by the expression of HBx [123]. Thus, by targeting various cellular pathways at various subcellular locations, these data may indicate that HBx expression plays a role not only in the pathogenesis of HCC but also in its development.

### 1.8. Regulation of the HBV HBx Gene Expression

**Figure 5:**
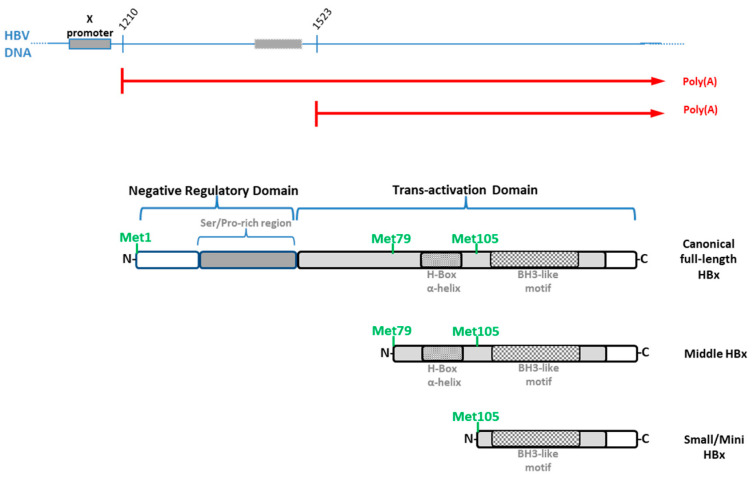
HBV HBx gene expression is controlled by both alternative translation and transcription initiation. At the top, HBV HBx DNA where the X promoter and the embedded intragenic promoter are shown. TSSs for HBV nucleotides 1210 and 1523 are shown [132] as well as the predicted HBx mRNAs, corresponding to XI and XII promoter transcription, in red (Supplementary Table S1). Below, the canonical HBx protein as well as the two smaller, divergent N-terminal isoform proteins: middle and small (mini) HBx [142].

Transcription of the canonical HBx gene is thought to be regulated by the X promoter (nt 1240 to 1375), located upstream of the transcription start site (TSS) (Figure 5).

The minimal promoter sequence overlaps the 3′-end of enhancer I (EnhI; nt 970 to 1240) [124,125]. The EnhI/X promoter is responsive to host factors, such as C/EBP, HNF1, HNF3, and CREB/ATF2, that control its transcriptional activity [38]. However, the existence of smaller HBx isoform proteins in vitro has suggested that the expression of HBx gene products might be regulated by either alternative translation or transcription initiation (Figure 5). Importantly, the viral HBx mRNA molecule bears two well-known internal in-frame translation initiation codons at the positions of AUG2 (Met79, 75 Aa, and 8.6 kDa) and AUG3 (Met105, 49 Aa, and 5.9 KDa), and these positions are highly conserved across HBV genotypes, although with different Kozak consensus sequences for translation initiation (Figure 5, Supplementary Table S1).

A mechanism by which a single gene has multiple TSSs is known as alternative transcription initiation. Under this mechanism, the transcription of the gene can begin from one of a few TSSs, and from various alternative core promoters [31,126]. Using alternative promoters, eukaryotes can expand the transcriptome and proteome diversity. In the case of human genes, more than 50% of the reading frames utilize alternative promoters [31,126,127,128,129,130].

Alternative transcription initiation in the regulation of HBx gene expression has been the subject of several studies. Smaller mRNAs bearing diverse 5′-ends upstream of the AUG2 initiation codon have been reported, and these transcripts could express HBx proteins after transfection into cultured cells (Figure 5) [131]. In line with this, by single-nucleotide resolution of HBV mRNAs, in addition to the canonical HBx mRNA, a shorter transcript with a peak position between the initiation codons AUG1 and AUG2 has been identified [132]. On the other hand, a ChIP-Seq methodology on viral cccDNA found a peak of histone marks associated to active promoters (H3K4me3 and H3K27ac) which were located within the HBx gene. This might imply the occurrence of an intragenic promoter between the AUG1 and AUG2 initiation codons [133]. Therefore, there is consistent evidence suggesting that alternative transcription initiation might also play a role in HBx regulation (Figure 5).

### 1.9. HBV Canonical HBx Protein and Roles on the cccDNA Intermediate

The nuclear HBV replication intermediate cccDNA is organized as a mini chromosome with regularly spaced nucleosomes containing histone and non-histone proteins [134,135]. Both core and HBx proteins can bind to the cccDNA and alter its structure [136,137], although the core protein is not required for the transcriptional regulation of the cccDNA [138]. Several labs, including ours, have described how histone post-translational modifications (PTMs) are associated with the cccDNA and can regulate viral transcription [4,137,139,140,141,142,143]. Methylation of histone H3 lysine 4 and hyperacetylation of histones correlate with active transcription, while methylation of histone H4 on arginine 3, methylation of histone H3 on lysines 9 and 27, and hypoacetylation of histones are all modifications that correlate with repressed transcription [144]. Importantly, chromatin-modifying enzymes that establish the modifications are engaged with the cccDNA. Histone variants are another player in the HBV viral transcription, as illustrated by H3.3 which is assembled into the cccDNA and activates transcription [141].

Importantly, the HBx protein regulates the recruitment of chromatin-modifying enzymes, modulating the cccDNA chromatin landscape. Indeed, in the presence of HBx protein, the cccDNA is in an active chromatin state, promoting HBV expression and production of viral progeny [137,139,140]. In contrast, in the absence of HBx, the cccDNA is in an inactive chromatin state [80,137,139,145]. Finally, HBV HBx-deficient infection resembles the occult infection observed in clinical cases; low transcriptional activity and persistence of the viral DNA [146], suggesting that the cccDNA is in an inactive chromatin state. It is worth noting that the occult HBV infection is predominantly caused by the HBV genotype H [13], whose infection is characterized by mild symptoms and minimal cases of acute and chronic liver diseases [9,10,11]. In several studies, HBV genotype H isolates replicated at low levels in culture cells [147]. Importantly, by amino acid sequence alignments of HBx from different genotypes and hosts, we previously showed that sequences corresponding to HBx genotype H do not exhibit the phylogenetically conserved AUG3 (Met3). Instead, most HBV genotype H isolates exhibit a codon encoding for a threonine, which means that this genotype is probably not expressing the HBx small isoform protein [142]. Whether this fact is related to the different clinical course of the infections caused by HBV genotype H is still unknown. Thus, it would be interesting to investigate the chromatin landscape of genotype H, and the relevance of the point mutation targeting the AUG3 codon in the HBx reading frame.

### 1.10. An Unusual Protein Variant of HBV HBx: HBV Whole-X (HBVwx)

**Figure 6:**
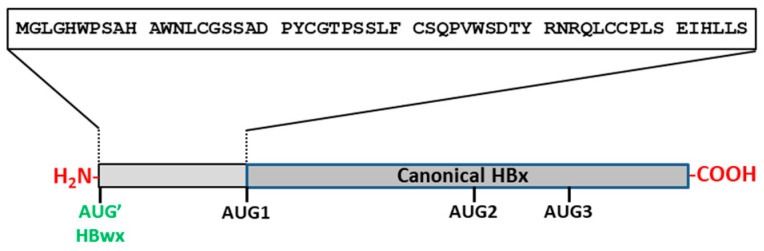
Scheme of the predicted residue sequence of the HBwx protein. Canonical HBx protein is shown with an N-terminal extension of 56 amino acid residues corresponding to the HBwx.

In 1990, Loncarevic et al. reported the finding of a unique HBx variant in five HBV DNA replication-competent clones isolated from HCC patients [148,149]. All these variants included an N-terminal extension of 56 amino acid residues in comparison to the canonical protein (Figure 6).

The reading frame of this variant protein, which has a length of 630 base pairs and 210 amino acid residues, was designated as HBV whole-X, HBwx, or preX [149]. The whole-X reading frame was found only in HCC patients from Asia and of Asian descent, and it was present only in HBV genotype C (adr isolates) samples, which is the genotype most closely associated with HCC [150]. Due to an UUG to AUG point mutation in the HBV DNA clone (e.g., GenBank X52939.1), these isolates have an extra in-frame AUG codon located 168 nucleotides upstream of the canonical HBx AUG1 and it enables translation of the HBwx/preX reading frame (Figure 6). The nature of the HBV mRNA that serves as a template for the HBwx/preX isoform variant is unknown.

Research on HBwx/preX has been developed using different approaches. With a yeast two-hybrid analysis, Yang et al. [151] demonstrated the interaction between HBwx and hepatoproteins, including fetuin B, UDP glycosyltransferase 1 family-polypeptide A9, mannose-P-dolichol utilization defect 1 (MPDU1), fibrinogen-B β polypeptide (FGB), and transmembrane 4 superfamily member 4-CD81 (TM4SF4). These findings led the authors to hypothesize that HBwx protein may influence the progression of hepatocarcinoma by altering the signal transduction of protein–protein interactions in liver cells [151].

In another publication, proteomic analyses investigated protein expression in cells expressing HBwx, compared with cells expressing canonical HBx [152]. A total of 203 proteins were found to interact with HBwx, of which 149 were HBwx interactors only, whereas the remaining proteins also interacted with HBx. Of these proteins, 73% (149/203) are implicated in the development of cancer. Gene ontology analyses revealed that HBwx- and HBx-interacting proteins belong to different pathways [152]. Whereas HBwx-interactors are primarily implicated in biosynthetic activities such as glycolysis, cell-cycle functions, and protein folding, canonical HBx interactors are involved primarily in angiogenesis, viral transcription, and cell adhesion. Raf-1 kinase inhibitory protein (RKIP) is one of the HBwx-regulated factors, widely documented to be directly related to HCC development.

Zhang et al. [153] analyzed the expression of HBwx in liver tissues of patients with HBV-related HCC and its effects on cell proliferation, apoptosis, and oncological profiles compared with those of canonical HBx. Seventy-two percent of the liver tumor tissue samples expressed HBwx, which was primarily localized in the nucleus. Both in vitro and in vivo cell growth, cell invasion, and migration were all positively influenced by HBwx. On the other hand, in vitro investigations showed that cells expressing HBwx differed in parameters such as cell proliferation, cell cycle, and cell apoptosis from those expressing the canonical HBx protein. Furthermore, they confirmed that the RKIP-p-ERK pathway played a role in the development of tumors associated with HBwx. These findings offered strong support for the role of HBwx in hepatocarcinogenesis [153].

### 1.11. Prior Findings Revealing the True Paradigm of the Canonical HBx and Isoform Proteins

It has been long recognized that the canonical HBx reading frame bears two internal in-frame translational start codons at AUG2 and AUG3, which may drive alternative translation initiation to produce two smaller distinct N-terminal isoform proteins (Figure 5). However, as briefly mentioned above, there are different findings which point out that alternative transcription initiation might also be involved in the control of the expression of HBx isoform proteins. Below, we review and describe most of the available evidence. Data on the occurrence of the HBx isoform proteins have been collected since 1991.

In a 1991 publication by Balsano [154], both full-length and truncated versions of HBx were described to be capable of transactivating the c-Myc proto-oncogene. The ability of the HBV HBx protein to transactivate genes, such as the HBV HBx itself and core promoters, HIV-1 and HIV-2 LTRs, and the interferon regulatory sequences, was known by that time. In this report, they stated that HBx can also affect the expression of the endogenous c-Myc gene as well as extrachromosomal transfected c-Myc regulatory sequences. Moreover, they also established that the full-length HBx protein did not impact the stability of the c-Myc mRNA and that the protein had a role in enhancing the transcription of the c-Myc gene. Interestingly, they also found that the production of a functionally active HBx protein required the first AUG codon of the HBx reading frame. At the C-terminus, the domain between residues 103 and 117 was crucial for the full-length HBx functionality (Figure 5). They stated that given that the integration of HBV DNA frequently results in truncation of the HBx reading frame in its 3′-end (as was shown by the sequencing of numerous cloned HBV integrants), the ability of several smaller HBx proteins to retain their transactivational capabilities was remarkable. The authors of this study proposed several possible mechanisms for producing different smaller HBx proteins. They noted that translation initiation might start at either the AUG1 or at one of two downstream in-frame AUG codons in the HBx coding sequence by alternative translation initiation (Figure 5). Significantly, this is the very first time that the idea of different HBx isoform proteins from a single HBx reading frame is ever mentioned in a scientific report of the field. It happened over 30 years ago [154].

In 1992, Kwee et al. [155] found that the canonical HBx protein (p17, 17 kDa) and the two distinct N-terminal isoform proteins (p8, 8 kDa, and p6.6, 6.6 kDa) (Figure 5) are indeed functionally different. They tested class II (RNA polymerase II)- and class III (RNA polymerase III)-transcribed promoters, finding that class III-transcribed promoters could be transactivated by each distinct HBx isoform protein; however, in class II promoters HBx isoforms acted differentially, depending on the promoter: the simian virus 40 enhancer/early promoter required the presence of the two HBx isoforms, p6.6 and p17. In contrast, only the individual full-length p17 protein was required for an NF-κB-dependent promoter. These findings seem to suggest that the various HBx isoforms can cooperate to cross-regulate their functions.

Additional analyses revealed that, at the C-terminus, a region of the HBx protein downstream of residue 118 was necessary for the activation of both class II and class III promoters, which is present in all HBx isoforms. It was shown that when the p6.6 HBx isoform cannot be expressed due to an AUG3 (Met) to GUG (Val) mutation in the full-length HBx reading frame, the activation of the SV40 enhancer/early promoter is abolished. The transcomplementation of p6.6 HBx isoform could revert to the activation of the SV40 enhancer/early promoter. These analyses brought profound attention to the crucial function of the HBx protein’s C-terminal region, particularly the p6.6 isoform. In summary, for the first time, this study characterized the in vitro activities of the canonical and two divergent N-terminal HBx isoform proteins (Figure 5) [155].

In 1993, Nakatake et al. [156] performed mutational analyses of HBx in-frame AUGs showing that transactivator protein products are generated by internal translation initiation. Importantly, they showed that an HBx mutant construct bearing stop codons at codons 118 or 130 did not transactivate the HBV promoter, whereas a mutant plasmid bearing a stop codon at the eighth codon retained a good amount of transactivation activity. On the other hand, an individual HBx construct with a UAG (stop codon) in place of the first AUG codon (with the other two AUGs intact) could retain one-third of the wild-type transcriptional activity. Significantly, these findings suggested that the C-terminal half of the HBx gene bears a critical portion of the potential to transactivate, thus one or both proteins translated from the AUG codons at positions 79 (AUG2) and 103 (AUG3) may reflect this activity (Figure 5). Their findings are consistent with the idea that the carboxy-terminal half of the HBx protein contains the domains that are most crucial for the transstimulatory function of HBx.

Finally, and based on their own data [156], the authors raised apprehensions regarding the use of HBV HBx-deficient constructs bearing mutations contained in the first half of the protein (at the N-terminal region). They proposed that the most suitable HBV HBx-deficient mutant is that which has none of the three AUG codons and has, therefore, been made incompetent for any HBx isoform proteins to be produced.

In 1994, the existence of short HBV HBx gene transcripts produced from an intragenic X promoter was demonstrated by Zheng et al. [131], by specific primer extension analysis of RNA isolated from HBV-transfected hepatoma cells. They found several HBx gene transcripts with diverse 5′-ends and shorter lengths than those reported in the literature. These shorter transcripts would include the AUG2 of the HBx reading frame and were not degradation products of the full-length HBx gene transcript.

They also demonstrated that the sequence that contains the segment upstream of the AUG2 of the HBx reading frame has an “embedded” promoter. In addition, this promoter would be functional even if there were no additional viral enhancers or promoters nearby. In their article, they suggested renaming the new X promoter as the small X (SX) promoter and renaming the upstream (canonical) HBx promoter as the large X (LX) promoter (Figure 5).

They then created an expression plasmid with the HBV DNA fragment spanning the AUG2 codon and the 3′-end of the HBx reading frame, under the transcriptional control of the SV40 enhancer region (and, so, it cannot express the full-length HBx protein). After that, they ran two different analyses. First, they transiently transfected this plasmid into Huh-7 cells, observing cytoplasmic staining of the cells. This finding supported the translation of smaller HBx isoforms from these transfected constructs. Second, co-transfection of cells with the same plasmid was used to assess the transactivation of the adenovirus reporter VA1 gene construct. The translated smaller HBx isoforms significantly activated the viral VA1 promoter. Accordingly, functional gene products with the distinctive transactivation properties of AUG2 (middle) and/or AUG3 (smaller) HBx proteins were generated from the short X transcripts [131]. However, it was not determined whether the middle and/or the small HBx isoforms were produced in these experiments (Figure 5).

In 2003, Leach et al. [157] investigated the effects of full-length HBx and the smaller alternative translation initiation X proteins on the expression levels of p21 (p21Cip1, alternative p21WAF1) and p27 (p27Kip1) cyclin kinase inhibitors (CKIs). Since HBx has both pro- and anti-proliferative effects, they also analyzed their impact on cell proliferation.

While low dosages of the canonical HBx or AUG2 protein led to increased p21/p27 expression, higher levels decreased p21/p27 expression. DNA synthesis decreased in the presence of elevated p21 and p27 protein levels and low expression of either canonical HBx, AUG2, or AUG3 proteins (Figure 5). In contrast, higher levels of AUG3 isoform expression led to increased p21/p27 expression and reduction of cell DNA synthesis. The canonical HBx protein inhibited this effect, suggesting that the full-length X protein has a dominant effect over the transcriptional control mediated by the smaller HBx proteins. Therefore, CKI expression and cell proliferation are controlled by the relative levels of canonical HBx, AUG2, and AUG3 expression. As a result, these findings might also help to explain the previously disparate impacts of HBx expression on cell proliferation [157].

In 2015, the first genome-wide map of histone PTMs associated with the viral cccDNA obtained from de novo infected HepG2 cells, primary human hepatocytes, and HBV-infected liver tissue was published by Tropberger et al. [158]. In these analyses, the histone PTMs associated with either active or repressive transcriptional states in the cccDNA were investigated.

Low levels of repressive histone PTMs were found, even at silent HBV promoters, whereas active histone PTMs were enriched at specific locations within the HBV genome. Data indicated that the regulatory EnhII/BCP region lacked nucleosomes. This region was then followed by a peak of active histone PTMs, resembling the pattern observed at the TSSs of many cellular transcribed genes. They did not discover similar peaks at the TSS of preS1, preS2, or HBx viral reading frames. Thus, the HBV regulatory EnhII/BCP region is probably the more active promoter in de novo infected HepG2-NTCP1 cells. On the other hand, they established that there are two discrete peaks of active promoter marks (H3K4me3, H3K27ac, and H3K122ac) within the HBx gene body, which are particularly pronounced in HBV-infected primary human hepatocytes and HBV-positive liver tissues [158] (Figure 5). These results consistently suggested the presence of an embedded intragenic promoter in the HBx gene and the possible transcriptional generation of smaller HBx isoform proteins.

A report detailing the mapping of HBV promoters was published in 2016 by Altinel et al. [132]. They developed the HBV TSS map utilizing human liver, HCC, and whole blood from HBV-positive patients, as well as experimental HBV replication systems. This crucial study was developed by cap analysis of gene expression (CAGE) at a single-nucleotide resolution.

They identified a faint peak corresponding to a segment of the canonical X promoter, upstream of the AUG1 of the HBx reading frame (peak 10, nt, 1210–1211). Importantly, they found a more pronounced peak (peak 11, nt, 1523–1524) between the AUG1 and AUG2 codons (Figure 5). They stated that this might result in the translation of a shorter HBx protein, when the highly conserved AUG2 is translated [132].

On the other hand, in addition to peaks 10 and 11, a large peak was found at approximately bp 1250, upstream of the first AUG, which may help with the expression of the canonical HBx protein (mainly detected in samples from HepG2.2.15 cells). Together, these results suggest that HBV may use several distinct promoters and different transcripts for the HBx isoform proteins depending on the cell environment and replication conditions [132].

Stadelmayer et al. introduced a fully new technique in 2020 [133], making it simpler to detect the entire spectrum of viral RNAs. They developed an HBV full-length 5′ rapid amplification of cDNA ends (5′-RACE) approach with which they were able to quantify and describe the entire viral RNA spectrum in both cell culture and chronically infected patient samples. It is interesting to note that they also investigated the kinetics of how various viral RNA types are expressed throughout infection. For example, they found that the HBx mRNA is the earliest transcript detectable, as early as 8 h post-infection in both HepG2-NTCP cells and PHHs. Importantly, both cells produced short types of the HBx mRNA transcripts. Consistently, sequencing the 5′-RACE amplicons showed significant diversity of TSSs on HBx transcripts expressed in either HepG2-NTCP cells or PHHs. These transcripts can be classified into two types: (i) “canonical” transcripts that start upstream (TSSs nt 1243 to nt 1338) of the first AUG of HBx and probably code for the full-length HBx protein and (ii) “shorter” transcripts that start downstream of the first AUG of HBx and probably code for a shorter version of HBx (Figure 5). They also identified capped and uncapped HBx viral RNAs present in viral particles [133].

A work using multiomics analyses to study HBV–host interactions throughout the life cycle of HBV was published in 2021 by Yuan et al. [159].

In cultivated hepatoma cells, they used the recently established Cre/loxP-mediated recombination method to continually generate recombinant HBV covalently closed circular DNA (rcccDNA) [160]. Secreted HBcAg was detected by an immunofluorescence assay 72 h after co-transfection of progenitor plasmids of recombinant cccDNA (prcccDNA) and pCMV-Cre. The “HBV-loaded cells” were subsequently utilized to assess cellular models of HBV replication and gene expression, RNA-sequencing ribosomal profiling (Ribo-seq), and quantitative mass spectrometry analyses.

In the Ribo-seq analysis, one peak showed a previously unidentified translational event. It was found that the HBx reading frame encodes more than a single protein, since ribosome footprints were concentrated on mRNA bearing a conserved AUG (AUG2) codon, indicating a possible internal in-frame translation initiation site (Figure 5). Thus, a shorter version of the HBx protein (named “HBxZ” [159]) corresponding to HBx AUG2 or HBx Middle is produced by the alternative translation start event.

An HBx AUG2 to CUG2 mutation in the HBV genome was performed and compared with that of the parental WT HBV genome after viral replication. The results indicated that HBxZ (HBx Middle) expression effectively reduced the expression of the HBV genes, and the authors proposed that HBxZ limits HBV protein expression in host cells [159].

In 2021, Hernandez and the members of our lab published a study characterizing the roles of the HBx isoform proteins during viral replication [142]. In this study, we initially demonstrated that the three distinct HBx isoform proteins are indeed produced from the ectopically expressed HBV HBx gene. The names XF (full-length), XM (middle-length), and XS (short-length/mini HBx) isoform proteins were given to these polypeptides (Figure 5). We demonstrated through mutagenesis that each of the three isoform proteins was synthesized independently from the HBx reading frame and that, upon overexpression, comparable quantities of them were produced.

Following this, the subcellular localization of each HBx isoform protein was investigated. The canonical HBx protein has distinct subcellular localizations depending on its expression level. The protein was mainly nuclear at low expression levels but mostly cytoplasmic at high expression. The HBx XF isoform was mostly restricted to the nuclear and nucleocytoplasmic compartments. It is interesting to note that the XM isoform protein and the canonical HBx protein behaved in opposite ways; at low expression levels, the XM isoform protein was mostly nuclear, but at high expression levels, it was mostly cytoplasmic. Enigmatically, the HBx XS (mini HBx) isoform protein remained cytoplasmic at different levels of protein expression. Therefore, we found out that the different HBx isoform proteins exhibit a variety of intersecting subcellular localizations which might indicate the various functions that are ongoing during HBV genome replication [142].

Analysis of the two smaller HBx isoform primary sequences uncovered that the HBx XM isoform bears an intact transactivation region (residues 50 to 154) but lacks the negative regulatory domain, which consists of residues 1 to 50 (Figure 5). The N-terminus of HBx protein contains a phylogenetically conserved but disordered and unstructured region, which includes a Ser/Pro-rich region [94]. The terminal regions of isoform proteins frequently perform regulatory and signaling functions. By selectively altering the profile of disordered regions they express, isoform proteins modify their interaction networks [161,162,163]. As a result, for example, the content of disordered regions in the various p53 isoform proteins differs, which is linked to either the establishment or abolishment of interactions with various factor partners [164] (Figure 4).

**Figure 7:**
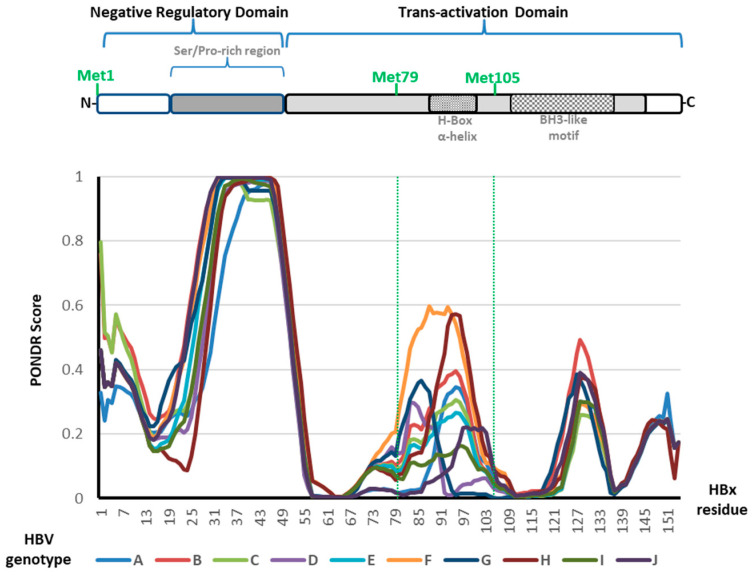
HBV HBx multigenotype amino acid sequence analyses of disordered regions. Amino acid sequences of HBx proteins from reference genotypes were run in the web server Predictor of Natural Disordered Regions (PONDR VL-XT) [165,166] for each of the ten HBV genotypes (genotypes A to J) [167]. Results using default parameter-based analysis are depicted.

For these reasons, we utilized a well-known web server, Predictor of Natural Disordered Regions (PONDR VL-XT) [165,166], to conduct analyses of predicted disordered regions of the full-length amino acid sequence of the canonical HBx protein. For each of the ten HBV genotypes (genotypes A to J) that have been described, we used one reference genome sequence [167]. Results using default parameter-based analysis are depicted in Figure 7.

Importantly, all the HBx protein sequences from the various HBV genotypes have features in their profiles that are equivalent to one another. Amino acid sequences of HBx from the different genotypes show similar tendencies outlining equivalent characteristics. We found two primary disordered sections in two restricted regions of the protein across genotypes (Figure 7): (i) residues between 25 and 55, which include the Ser/Pro-rich region, represent the highest peak of sequence disorder and (ii) residues between 79 and 104, which contain the H-box, a promiscuous alpha-helix. This second disordered region is enclosed within the AUG2 to AUG3 segment. As a result, the N-terminus of each HBx isoform is surrounded by disordered regions; the HBx XM isoform lacks the Ser/Pro-rich region but retains the second disordered section. However, despite only having less than half of the HBx transactivation domain, the HBx XS isoform protein contains the alpha-helix BH3-like (BH: Bcl-2 homology) motif necessary for interaction with the anti-apoptotic protein Bcl-2 [100,168] (Figure 7). Consequently, given that the HBx isoforms have a variety of disordered sections, functional domains, and subcellular locations, one might anticipate significant changes that can affect their activities during protein expression and viral replication.

**Figure 8:**
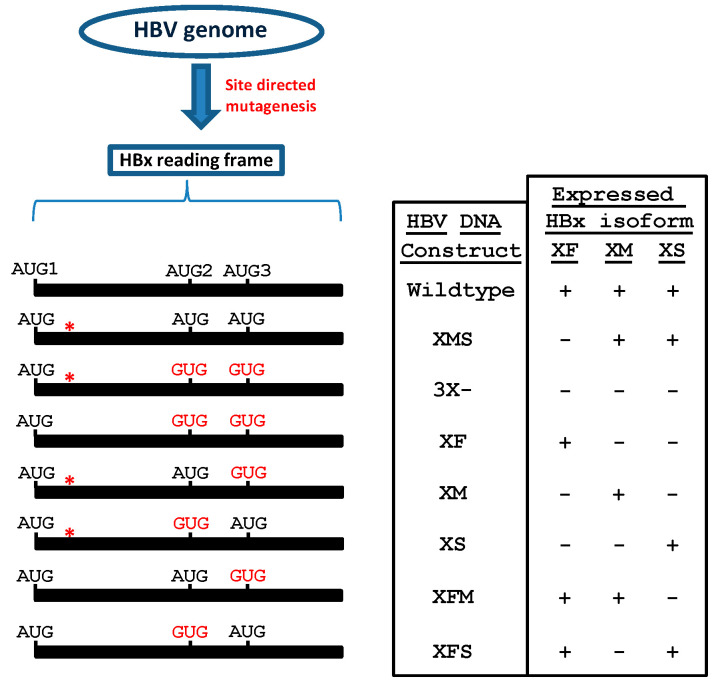
Outline of the site-directed mutagenesis carried out on HBV DNA backbone for the individual or combined expression of the HBx isoform proteins during viral replication. Internal in-frame translation initiation codons at AUG2 and AUG3 in the HBx reading frame were replaced by a GUG codon (valine) to prevent translation initiation at the site. To prevent the translation of the full-length HBx protein, a stop codon was introduced in the eighth codon of the HBx reading frame and it is indicated by an asterisk (*). Names of each HBV DNA construct are given in right panel as well as the expressed HBx isoform protein in each case [142].

By introducing several point mutations directly into the HBV HBx DNA, we were able to target the expression of all HBx isoform proteins, either individually or in any possible combination, and we were able to investigate the HBx isoform during viral replication [142]. Figure 8 outlines the different HBV constructs, as well as the anticipated isoform proteins expressed by each viral construct.

When the HBV genome was targeted to simultaneously eliminate the expression of all three HBx isoforms (construct HBV 3X-, Figure 8), levels of cytoplasmic DNA viral core particles (cytDNA) were lower than those of the XMS HBV genome. Our HBV XMS viral construct is comparable to the viral construct that has been utilized as a typical strategy “to abolish HBx expression” and as an “HBV HBx-deficient” construct [79,81]. By abolishing the expression of all three HBx isoforms, the transfection with the HBV 3X- construct reduced the presence of the cytDNA marker to background levels. Neither XF nor XM alone were sufficient to restore WT levels of HBV replication, however, cytDNA levels were similar to those of the WT HBV genome when we transfected the HBV DNA that only expressed the HBx XS isoform protein (Figure 8).

We also looked for HBsAg and HBeAg, which are secreted markers of acute and active HBV replication. The expression of the XS isoform resulted in the highest amount of HBsAg secretion, 80% of that of WT HBV. On the other hand, the HBeAg value of the HBV XS genome was identical to that of the WT HBV. As a result, the released viral antigens from the WT HBV genome and the HBV XS genome were nearly equivalent. Together, these findings suggest that the HBV viral cycle strongly depends on the HBx XS isoform.

We investigated whether the HBx XS (mini HBx) isoform contributes to the formation of an active HBV chromatin state. First, we investigated if certain HBx isoform proteins can bind to the viral cccDNA nuclear intermediate. For this, we co-transfected constructs that each expressed HBx XF, XM, or XS isoform proteins tagged at their C-terminal with GFP protein with the WT HBV DNA genome in HepG2 cells. We performed chromatin immunoprecipitation (ChIP) analyses against GFP protein. Interestingly, of the different individual HBx isoform proteins that were assessed, only the HBx XS isoform was found at the viral core promoter. Next, we looked at the histone PTMs linked to the cccDNA viral nuclear intermediate. HepG2 cells were transfected with either the WT HBV DNA, the HBV 3X- construct, or the HBV XS construct and we conducted ChIP experiments against either the active H3K4me3 mark or the repressive H3K9me2 with a focus on the viral core promoter. Both the WT and HBV XS cccDNA contained more of the transcriptionally active H3K4me3 mark, whereas the mutant HBV3X- genome contained more of the transcriptionally inactive histone repressive H3K9me2 mark. The transcriptional activity of the HBx proteins is in line with these findings. Thus, because the HBx XS (mini HBx) isoform protein is capable of binding to the viral core promoter DNA and this isoform is enough to generate an active HBV chromatin state, HBx XS (mini HBx) isoform protein is then crucial to fully recapitulate HBV replication.

## 2. Conclusions and Future Directions

The number of known divergent N-terminal isoform proteins is growing, and there are examples from varied eukaryotes such as mammals, plants, and yeast [26,27]. In photosynthetic eukaryotes, the ATP sulfurylase (ATPS) enzyme isoforms show a differential subcellular localization in both plastids and cytosol [169]. In the yeast *Saccharomyces cerevisiae*, the two isoform proteins of glutathione reductase (GR) generate either mitochondrial or cytoplasm pools of the enzyme [170]. Moreover, in animal cells, the generation of isoform proteins takes place on gene products such as the polypyrimidine tract-binding protein (PTBP1), mitochondrial antiviral-signaling protein (MAVS) [171,172], Runt-related transcription factor 1 RUNX1 [173], p53 [174], caspase-2 [175], stress-activated protein kinase MK2 (stress/p38^MAPK^-activated protein kinase MK2) [176], glucocorticoid receptor (GR) [177], tumor suppressor phosphatase and tensin homolog (PTEN) [178], and human c-Myc proto-oncogene [179]. For HBV HBx, the occurrence of N-terminal divergent isoform proteins will modify the canonical, multifunctional, and unique HBx protein paradigm by changing the concept into different isoforms, each performing distinct overlapping roles at different intracellular sites during HBV genome replication. We strongly advise researchers to pay extreme attention when performing site-directed mutagenesis in either the individual HBx reading frame or the HBV HBx backbone and consider the targeting of unexpected changes carried out not only in the canonical HBx reading frame but also in the smaller HBx isoforms.

Unfortunately, after far more than three decades of research on HBV HBx, not only the exact mechanism of action but also the precise regulation of its many different functions remain elusive. However, HBx is considered to be essential for HBV genome replication, as discussed above. Table S2 (Supplementary Materials) summarizes the main characteristics of the canonical and HBx isoform proteins to date.

Even though this subject has been comfortably ignored for decades, this re-discovery of the roles of HBx isoform proteins not only in HBV replication but also possibly in cell pathogenesis will make it even more complex to individualize their biological roles in infected cells [142]. HBx has no homologous protein or known functional relatives. However, different regulation pathways remain unexplored. For example, for the hepatitis D virus antigen (HDVAg), two main isoform proteins are functionally regulated by post-translational modifications [180]. On the other hand, the roles of the chromatin and non-chromatin histone isoform proteins are tightly regulated by post-translational modifications [181]. Thus, we finally ask, are the roles and subcellular localizations for the canonical and HBx isoform proteins controlled, for example, by post-translational modification, adding different functional groups to the different protein backbones [182]? Regarding this, we have recently shown that the subcellular localization of the canonical HBx might be regulated by phosphorylation on phylogenetically conserved amino acid residues [110] and, therefore, this is a subject that we certainly should assess next.

## Data Availability

No new data were created.

## Supplementary Materials

The following are available online at https://www.mdpi.com/article/10.3390/biomedicines11061674/s1, Table S1: Predicted proteins from HBV-encoded reading frames based on reliability of AUG initiation codons; Table S2: Main characteristics of the canonical and HBx isoform proteins.
