# Supplementary material for: Pre- and Post-Transcriptional Control of HBV Gene Expression: The Road Traveled towards the New Paradigm of HBx, Its Isoforms, and Their Diverse Functions
Source: Biomedicines. 2023 Jun 9;11(6):1674. doi: 10.3390/biomedicines11061674 (PMC10296101; doi:10.3390/biomedicines11061674)
Supplement: Supplementary file 1 [file biomedicines-11-01674-s001.zip › biomedicines-2402481-supplementary.pdf]

Table S1. Predicted proteins from HBV encoded reading frames based on reliability of AUG initiation codons[1].

mRNA[2]	Viral protein[3]	Identity to Kozak rule A/GXXATGG	Reliability[4]	ORF length (Aa)	Predicted molecular weight (kDa)	Sequence[5]
pg RNA	**canonical core protein**	GXXATGG	0.36	183	21	H_2N-**M1**-X64-**M2**-X26-**M3**-X90-COOH
	core isoform 1	tXXATGa	0.11	118	13.6	H_2N-**M2**-X26-**M3**-X90-COOH
	core isoform 2	AXXATGG	0.07	91	10.6	H_2N-**M3**-X90-COOH
pg RNA	**canonical P protein**	cXXATGc	0.91	843	94.2	H_2N-**M1**-X473-**M2**-X179-**M3**-X188-COOH
	P protein isoform 1	AXXATGc	0.31	369	41	H_2N-**M2**-X179-**M3**-X188-COOH
	P protein isoform 2	cXXATGc	0.29	189	20.8	H_2N-**M3**-X188-COOH
preC/C	**precore/core protein**	AXXATGc	0.56	212	24.2	H_2N-**M1'**-X28-**M1**-X64-**M2**-X26-**M3**-X90-COOH
	core isoform 1	tXXATGa	0.11	118	13.6	H_2N-**M2**-X26-**M3**-X90-COOH
	canonical core protein	GXXATGG	0.09	183	21	H_2N-**M1**-X64-**M2**-X26-**M3**-X90-COOH
	core isoform 2	AXXATGG	0.07	91	10.6	H_2N-**M3**-X90-COOH
**PreS1**	**canonical LS protein**	AXXATGG	0.95	400	43.7	H_2N-**M1**-X118-**M2**-X54-**M3**-X225-COOH
	MS protein isoform	GXXATGc	0.24	281	31	H_2N-**M2**-X54-**M3**-X225-COOH
	S protein isoform	AXXATGG	0.18	226	25.1	H_2N-**M3**-X225-COOH
**S**	**MS protein isoform**	GXXATGc	0.54	281	31	H_2N-**M2**-X54-**M3**-X73-**M3'**-X151-COOH
	S protein isoform	AXXATGG	0.18	226	25.1	H_2N-**M3**-X73-**M3'**-X151-COOH
	S-smaller protein isoform	tXXATGt	0.09	152	17.2	H_2N-**M3'**-X151-COOH
XI	**canonical HBx protein**	cXXATGG	0.53	154	16.5	H_2N-**M1**-X77-**M2**-X25-**M3**-X49-COOH
	middle HBx isoform	cXXATGG	0.08	76	8.6	H_2N-**M2**-X25-**M3**-X49-COOH
	smaller/Mini HBx isoform	tXXATGa	0.04	50	5.8	H_2N-**M3**-X49-COOH
XII	middle HBx Isoform	cXXATGG	0.16	76	8.6	H_2N-**M2**-X25-**M3**-X49-COOH
	smaller/Mini HBx isoform	tXXATGa	0.04	50	5.8	H_2N-**M3**-X49-COOH

[1] https://atgpr.dbcls.jp/, [33].
[2] mRNA considering each individual viral canonical reading frame.
[3] In bold, each viral canonical reading frame.
[4] Reliability is the likelihood that the estimate will be achieved accurately.
[5] Canonical Met are in red, and potential Met isoforms are in bold.

Table S2. Main characteristics of the Canonical and HBx isoform proteins.

	Name[1]	Amino acids[2]	Molecular weight, (kDa)[2]	Isoelectric point, (pI)[2]	Subcellular Location[1]		Individual effect on HBV replication[1,3]
					High abundance	Low abundance	
**Canonical HBx**	Wildtype	154	16.6	8.91	Cytoplasmic	Nuclear	Activating
**HBx XF**	Full-length	154	16.5	8.91	Nucleocytoplasmic/ Nuclear	Nucleocytoplasmic	Repressing
**HBx XM**	Middle	76	8.6	8.82	Nuclear	Cytoplasmic	Repressing
**HBx XS**	Small, mini	50	5.8	6.53	Cytoplasmic	Cytoplasmic	Activating

[1] As established in [142].

[2] Corresponding to HBx proteins from HBV subgenotype F1b, Genbank KM233681.1, 3215 bp [3, 4].

3 As established in [159].
